# Current literature support and opposition for therapeutic use of selenium supplementation in autoimmune thyroid conditions

**DOI:** 10.1016/j.bbrep.2025.102013

**Published:** 2025-04-17

**Authors:** Abhinav Nannapaneni, Victoria Wilson, Shahzaib Chughtai

**Affiliations:** Central Michigan University College of Medicine, United States

**Keywords:** Selenium, Selenium supplementation, Autoimmune thyroid disease (AITD), Graves' disease, Hashimoto's thyroiditis, Lymphocytic thyroiditis, Lymphomatous thyroiditis, Autoimmune hypothyroid, Autoimmune hyperthyroid

## Abstract

This systematic review investigates the correlation between selenium status and thyroid conditions, specifically focusing on autoimmune thyroid diseases (AITD) including Hashimoto's Thyroiditis (HT) and Graves' Disease (GD). After a meticulous analysis of 14 research studies, we explored the impact of selenium levels and supplementation on several thyroid-specific parameters and overall disease progression. Several studies indicated a notable inverse correlation between selenium deficiency and the prevalence and severity of AITD. In particular, HT patients showed consistent improvement with selenium supplementation, suggesting its potential as a therapeutic agent. However, the effect of selenium on GD was not as clear, with some studies indicating no significant correlation. These inconsistent effects demonstrate how nuanced AITD treatment must be. It also shows that selenium plays a varied role in thyroid health. The findings in this review serve as an example of how micronutrient intake is important in thyroid disease management. The nuances of these treatments support a need for more patient-centered and individualized treatment plans, which may be further helped by detailed research into proper dosing and administration strategies. Specific patient populations may benefit from selenium supplementation, though the variability in outcomes points to a need for vigilance in the clinical setting. Importantly, the ability to personalize supplementation depends on establishing a reliable method for detecting selenium deficiency or status. Future clinical guidelines should emphasize the necessity of a solid diagnostic framework that allows for precise monitoring, ensuring that selenium supplementation is appropriately justified and tailored to individual patient needs.

## Highlights

• Systematic review conducted to analyze the impact of selenium levels on the thyroid.
• Graves disease shows an association with selenium levels, while Graves ophthalmopathy does not.
• Hashimoto thyroiditis is associated with low plasma selenium.
• Review of levothyroxine treatment enhancement with selenium supplementation.
• Selenium may help reduce thyroid antibody groups, suggesting potential for autoimmune thyroid disease treatment.

## 1 Introduction

**Fig. 1:**
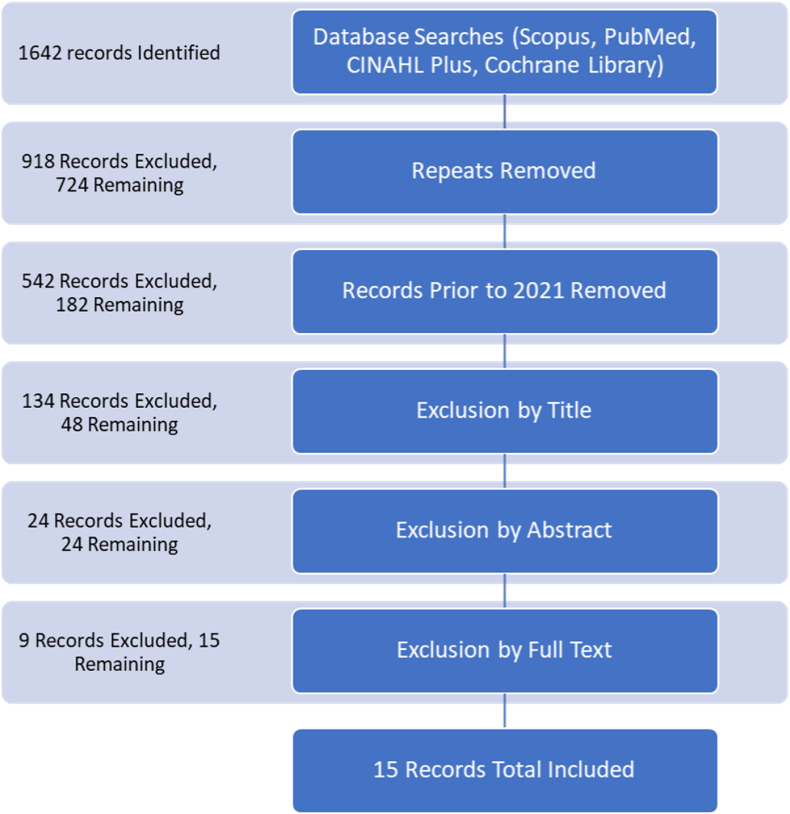
PRISMA flow diagram showing the filtering process of studies that were initially identified for review. Of these 15, 1 article was later retracted, leaving 14 in the final analysis.

The micronutrient selenium has been under investigation for some time now as a potential new treatment for several well-understood thyroid pathologies that contribute substantially to global morbidity from chronic health conditions. Selenium is currently understood to play a role in thyroid hormone conversion, as a part of the amino acid selenocysteine, which is the active amino acid in the catalytic sites of deiodinase enzymes [1]. This more modern understanding allows us to consider selenium deficiency as potentially implicated in thyroid pathologies and selenium supplementation as a potential therapeutic for patients dealing with such conditions, though the data on these is still limited and controversial. Reviews of these data in the past several years have found that selenium seems to be tied particularly to AITDs. AITDs are those conditions of the thyroid where the body's own immune system is implicated in the disease pathogenesis via production of autoantibodies that act erroneously at the thyroid, resulting in significant disruptions to its function. Manifestations of AITDs vary, depending on the specific target of the given elements (see Table 1, Table 2, Table 3, Table 4, Fig. 1).

**Table 1:** Compilation of studies focusing on Hashimoto thyroiditis that were used in the review and their respective features, including focus, findings, and variables.

Study	Overall Focus	Specific Thyroid Variable	Significance/Correlation	Selenium Status/Intervention
Cinemre et al. (2022)	HT Female patients	HT status	Significant (negative correlation, p = 0.002)	Selenium levels
Hu et al. (2021)	HT	(TPOAb levels)	Significant (negative correlation, p = 0.001)	Selenium Supplementation
Kryczyk-Kozioł et al. (2021)	HT Kraków female patients	HT status	Significant (negative correlation, p < 0.05)	Selenium supplementation
Wang et al. (2021)	HT	High/low-antibody groups (TGAb/TPOAb)	Significant (negative correlation, p < 0.05) High-antibody group: - TGAb 3 months (not significant, p > 0.05) 6 months (significant, negative correlation, p < 0.05). - TPOAb at 3 and 6 months (significant, negative correlation, p < 0.05) Low antibody group: - TGAb and TPOAb 3 and 6 months (not significant)	Selenium supplementation
Wu et al. (2022)	HT Shaanxi Province, China, specifically in Ningshan (low-Se area) and Ziyang (adequate-Se area)	HT status	Significant (negative correlation, p < 0.0001)	Selenium deficiency
Zheng et al. (2023)	HT	HT status	Significant (negative correlation, p < 0.0001)	Selenium intake
Summary			6/6 studies demonstrate significant negative correlations between HT and selenium.

**Table 2:** Compilation of studies focusing on GD that were used in the review and their respective features, including focus, findings, and variables.

Study	Overall Focus	Specific Thyroid Variable	Significance/Correlation	Selenium Status/Intervention
Halawa et al. (2022)	GD	GD/Graves Ophthalmopathy (GO) status	GD and selenium deficiency (significant, negative correlation, p < 0.05) GO (Graves ophthalmopathy) (not significant, p > 0.05)	Selenium status
Kravchenko et al. (2021)	GD Kyiv region patients	GD status	Significant (negative correlation, p < 0.001)	Selenium deficiency
Summary			2/2 studies demonstrate significant negative correlations between selenium and GD (not with GO).

**Table 3:** Compilation of studies focusing on autoimmune thyroiditis that were used in the review and their respective features, including focus, findings, and variables.

Study	Overall Focus	Specific Thyroid Variable	Significance/Correlation	Selenium Status/Intervention
Davchev et al. (2022)	AITD Bulgarian patients	Euthyroid AITD/Hypothyroid/Hyperthyroid	Euthyroid AITD + Hypothyroid (not significant) Hyperthyroid (significant, negative correlation, p < 0.001)	Selenium deficiency
Heidari & Sheikhi (2022)	AITD Population in iodine sufficient area in eastern Iran	AITD status	Significant (negative correlation, p < 0.001)	Selenium deficiency
Kryczyk-Kozioł et al. (2022)	AITD Kraków female patients	AITD- [INF-γ (Interferon γ)], [IL-1β (Interleukin 1β)]	Significant INF-γ decreases with treatment. (significant, negative correlation, p = 0.04). IL-1β increases with treatment (significant, positive correlation, p = 0.01)	Selenium supplementation
Vaivode et al. (2023)	AITD	AITD status	HT (significant, negative correlation, p < 0.05) GD (not significant)	Selenium level
Zake et al. (2021)	AITD Treatment naive Latvian patients	HT/GD/TPOAb levels	TPOAb levels (significant, negative correlation, p = 0.02) Patient Disease Status HT/GD (not significant)	Selenium plasma levels
Summary			5/5 studies demonstrate significant negative correlations between AITD and selenium

**Table 4:** Description of study which focused on thyroid conditions other than AITD, GD, or HT, that were used in the review and their respective features, including focus, findings, and variables.

Study	Overall Focus	Specific Thyroid Variable	Significance/Correlation	Selenium Status/Intervention
Mahmoudi et al. (2021)	Subclinical Hypothyroidism	Serum TSH Level & Serum TPOAb Level, as markers of subclinical Hypothyroidism status	Not significant (p > 0.05)	Selenium supplementation
Summary			1 study showed a non-significant correlation between thyroid condition and selenium

This is exemplified in the comparison between two of the most prominent examples of AITD, GD, and HT. In GD, thyroid-stimulating immunoglobulins (TSIs) bind to and activate the thyroid-stimulating hormone (TSH) receptors, producing a hyperthyroid condition. While on the opposite side, in Hashimoto's thyroiditis antibodies against thyroid peroxidase (TPO) and thyroglobulin (TG) - antithyroid peroxidase antibodies (TPOAb), and antithyroglobulin antibodies (TGAb) - leading to destruction of the thyroid tissue and resulting in a hypothyroid state. These are the conditions, for which the authors of works analyzed in this review, hope to examine the existing and potential roles of selenium.

For example, Zuo et al. in their 2021 review of 17 randomized controlled trials (RCTs), published between January 2000 and November of 2020, observed substantial reductions in free triiodothyronine (FT3), free thyroxine (FT4), and TPOAb in AITD patients who received any of a spectrum of “selenium-containing drugs” compared to those who didn't, across several clinical scenarios [2]. Similarly, Giammanco et al., corroborates these observations in their 2023 review where they describe finding that selenium administration along with anti-thyroid drugs appears to restore euthyroid condition in patients faster than the anti-thyroid drugs alone [3]. Though they do also note that the data surrounding dosing, duration, efficacy as a therapeutic, and safety/adverse effects of selenium as a potential supplemental treatment are limited and controversial. Deischinger et al. in their 2022 article similarly describe selenium supplementation as demonstrating “vast potential” for hypothyroid and AITD patients, but do also note a need for further studies on specific patient populations to inform endocrinology guidelines regarding its use [1].

While these reviews are promising, they do group quite a few clinically distinct pathologies – notably, for example, both hypo and hyperthyroid clinical states, as well as both autoimmune and non-autoimmune conditions - and report their results very generally. While these concerns are presumably by necessity, given the relatively limited number of studies in this area, there are clearly also extensive gaps in our information base surrounding when and how selenium supplementation could be used in the treatment regimen for patients with AITDs. Plus, they demonstrate a substantial need to differentiate between separate patient groups and clinical scenarios under the wider umbrella of AITDs as a group. The above reviews demonstrate clear opportunities for investigations into which compounds could be most beneficial, for which patient populations, and with what dose and schedule, among many others. Research in these areas is continuing to develop, with a notable uptick in the last three years (2021–2023), approximately, of projects investigating these more specific inquiries. The goal of this review is to compile some of these more recent data and evaluate their potential implications on the clinical use of selenium supplementation. Specifically, this project seeks to identify patterns in recent literature (2021–2023) pertaining to the potential use of selenium supplements in mitigating the effects of AITDs. Our intent is to analyze the current literature to answer the question: **Are there improvements – either individually reported or clinically quantifiable – in the condition of patients with thyroid conditions of autoimmune etiologies who have a higher dietary intake of selenium, either by supplementation or by food source selection - including passive selection, such as living in an environment with more selenium-rich soil, etc. – as compared to patients who have no supplementation or consume less selenium-rich foods?** In essence, we seek to correlate selenium status, particularly as related to selenium intake, with presentations of AITDs as an early step in mapping which AITD presentations and patient populations are likely to benefit from including selenium supplementation in their treatment. To this end:

Our Hypothesis: Patients with autoimmune thyroid conditions whose dietary intake of selenium is increased, instead of those with lower selenium intake, will have subjectively reported and/or clinically quantifiable improvements in the measures associated with their condition compared to patients with lower dietary intake.

Null Hypothesis: There will be no significant difference between the measures of autoimmune thyroid conditions experienced by patients who have increased dietary selenium intake compared to those experienced by patients who have a lower intake of selenium in their diet.

## 2 Methods

### 2.1 Variables

Because autoimmune thyroid conditions do vary in presentation and in the methodologies used to monitor their severity, it is necessary to define the variables named in our research question somewhat flexibly, in order to capture the variety of pathologies we hope to differentiate. The dependent variable/outcome is termed “improvements” above per this review's objective of evaluating potential connections between selenium intake and improved morbidity among autoimmune thyroid patients. To be operational in this investigation, the outcome variable must be defined in a manner that draws from the wide variety of measures used to diagnose AITDs and to evaluate their progression and severity. To this end, we've selected several categories of parameters which either appear extensively in the literature on thyroid conditions or are used clinically to evaluate patients dealing with these conditions. The categories selected are as follows.

a. Subjective Reporting: Any measure that relies on patient self-reporting, such as quality of life questionnaires, pain scales, documented patient interviews, symptom progression (eg. improvement in fatigue), and similar.
b. Thyroid-Specific Lab Values: Any thyroid-related value that can be monitored by bloodwork or similar analysis, such as antibody titers (eg. TPOAb), thyroid hormones (eg. TSH, TRH, T3, T4), and similar.
c. Measures of Oxidative Stress: Lab values or other indices that are not specific to thyroid disease but still inform the investigators regarding inflammation and disease progression, such as MDA, CRP, TOS, OSI, and similar.
d. Physical Exam Findings: Any item that can be observed clinically and quantified per the physician's judgement or standard clinical practice, such as changes to hair or skin, thyroid volume/goiter/nodules, clinical signs (Stellwag's, Kocher's, Möbius, etc.), clinical scales or scores (eg. CAS score), and similar.
e. Secondary Conditions: Any quantifiable monitoring of conditions that the patients experience as a known secondary result of their autoimmune thyroid condition, such as orbitopathy, obesity, depression, lethargy, obstipation, fatigue, and similar.
f. Other: Any measure chosen by the investigators that falls outside of the above categories – typically per the extreme specificity of the parameter being accounted for – such as HDL-DR expression in thyrocyte cultures, fine needle biopsies, selenoenzymes, etc.

∗ Anti-TG (Antithyroglobulin), TSH (thyroid stimulating hormone), TRH (Thyrotropin-releasing hormone), MDA (malondialdehyde), T3 (triiodothyronine), T4 (thyroxine), CRP (C-reactive protein), TOS (Thyroid oxidative stress), OSI (Oxidative stress index), CAS score (Clinical activity score for thyroid eye disease), HDL-DR (High density lipoprotein receptors)

Similarly, because the mechanisms of selenium as a micronutrient are not well understood, “higher intake of selenium,” discussed above as the independent variable in this review, must also be defined flexibly, again per the methods from the included studies. The categories selected to qualify studies for inclusion in this review – which were selected per their frequent appearances in the literature and their ability to be evaluated as independent variables in connection with our selected dependent variables - are as follows.

a. Higher dietary intake by active selection of selenium-rich foods, versus no intentional diet changes
b. Higher dietary intake by selenium supplementation, versus no supplementation
c. Higher dietary intake resulting from living in a selenium-rich area where the increased selenium in the soil impacts the amount of selenium consumed in produce, versus selenium-poor areas where the produce contains lower amounts
d. Higher measures of selenium in the body, such as serum selenium, selenoprotein P (binds selenium in the blood), selenoenzymes, and similar, versus lower measured values

## 3 Data collection

For this analysis, searches were conducted via PubMed, CINAHL, Cochrane Library, and Scopus databases using MeSH Terms and search terms defined in collaboration with and structured by CMED's medical Librarian. The search terms used are as follows.

### 3.1 PubMed search

("Selenium"[Mesh] OR "Selenium Oxides"[Mesh] OR "Selenium Compounds"[Mesh] OR "selenium"[tiab]) AND ("Thyroiditis, Autoimmune"[Mesh] OR "Graves Disease"[Mesh] OR "Hashimoto Disease"[Mesh] OR "Thyroiditis, Subacute"[Mesh] OR "Graves disease"[tiab] OR "Hashimoto∗"[tiab] OR "lymphocytic thyroiditi∗"[tiab] OR "lymphomatous thyroiditi∗"[tiab] OR ("autoimmune"[tiab] AND "thyroid"[tiab]) OR ("subacute"[tiab] AND "thyroiditi∗"[tiab]))

#### 3.1.1 CINAHL search

((MH Selenium) OR (MH "Selenium Compounds") OR (TI selenium OR AB selenium)) AND ((MH "Thyroiditis, Autoimmune") OR (MH "Graves' Disease+") OR (TI "Graves disease" OR AB "Graves disease") OR (TI Hashimoto∗ OR AB Hashimoto∗) OR (TI "lymphocytic thyroiditi∗" OR AB "lymphocytic thyroiditi∗") OR (TI "lymphomatous thyroiditi∗" OR AB "lymphomatous thyroiditi∗") OR ((TI autoimmune OR AB autoimmune) AND (TI thyroid OR AB thyroid)) OR ((TI subacute OR AB subacute) AND (TI thyroiditi∗ OR AB thyroiditi∗)))

#### 3.1.2 Cochrane Library search

([mh Selenium] OR [mh "Selenium Oxides"] OR [mh "Selenium Compounds"] OR selenium:ti,ab) AND ([mh "Thyroiditis, Autoimmune"] OR [mh "Graves Disease"] OR [mh "Hashimoto Disease"] OR [mh "Thyroiditis, Subacute"] OR "Graves disease":ti,ab OR Hashimoto∗:ti,ab OR ("lymphocytic" NEXT thyroiditi∗):ti,ab OR ("lymphomatous" NEXT thyroiditi∗):ti,ab OR (autoimmune:ti,ab AND thyroid:ti,ab) OR (subacute:ti,ab AND thyroiditi∗:ti,ab))

#### 3.1.3 Scopus search

(INDEXTERMS(Selenium) OR INDEXTERMS("Selenium Oxides") OR INDEXTERMS("Selenium Compounds") OR TITLE-ABS(selenium)) AND (INDEXTERMS("Thyroiditis, Autoimmune") OR INDEXTERMS("Graves Disease") OR INDEXTERMS("Hashimoto Disease") OR INDEXTERMS("Thyroiditis, Subacute") OR TITLE-ABS("Graves disease") OR TITLE-ABS(Hashimoto∗) OR TITLE-ABS("lymphocytic thyroiditi∗") OR TITLE-ABS("lymphomatous thyroiditi∗") OR (TITLE-ABS(autoimmune) AND TITLE-ABS(thyroid)) OR (TITLE-ABS(subacute) AND TITLE-ABS(thyroiditi∗)))

The results of the searches were compiled in Endnote, totaling 1642 originally, and repeats were eliminated, leaving 724 results total. From these, any records prior to 2021 were removed to focus on the time-frame specified by our research question, leaving 182 records. We then used Chat GTP 4 to preliminarily sort these records by title according to our include/exclude criteria, described below. Our include and exclude criteria were fed to the artificial intelligence (AI) as written - along with our variable definitions (as above) and some general instructions - to guide the sorting choices. We then checked each item on the AI-generated include and exclude lists individually to confirm whether we agreed with the sorting and to re-sort any choices we disagreed with. After this process there were 48 records remaining for the abstract review step. We excluded 24 more records during abstract review and then 9 more during full text review, leaving 15 records total to be included in this review. 1 of these 15 was found to have been retracted, leaving 14 in the final review. These steps are summarized in the PRISMA diagram under results. Our inclusion and exclusion criteria for the data collection described here were as follows.

### 3.2 Inclusion

- Autoimmune thyroid conditions of interest (GD, HT, etc.)
  o Including studies that focus exclusively on one or more pathology secondary to the thyroid pathology (must be clearly identified as and understood to be a result of the thyroid pathology)
- Studies on diseases that are specific to selenium deprived populations, such as myxedematous cretinism, Kashin–Beck disease, and Keshan disease, as these will assist in articulating selenium's overall role in nutrition.
- Intervention in the study was a compound containing selenium (ie. Study must measure and evaluate selenium and its impact on the above conditions.
- Studies where variables and definitions fit the criteria outlined.
- Only Human subjects studies

### 3.3 Exclusion

- Exclude articles older than the last 3 years (prior to 2021)
- Exclude studies focused on pregnant or postpartum patients
- Exclude studies focused on patients with other chronic comorbidities (eg. Down's syndrome, diabetes, etc.)
- Excluded studies on patients experiencing diseases that have not shown any physiological or epidemiological link to selenium in the literature
- Exclude studies on patients with non-Autoimmune Thyroid diseases
- Exclude other conditions that are currently being investigated separately for a relationship to selenium intake, (such as diabetes and certain cancers.)
- Exclude Non-English papers and unpublished studies
- Exclude animal studies.
- Excluding studies where the interventions were combination therapy treatments that included selenium, rather than selenium compounds alone.
- Exclude studies where variables/definitions/conditions/etc. Do not fit the criteria and parameters outlined in the research question definitions.

During the review process, there were three revisions to these criteria, per the results of the literature search. The first was that the inclusion point on diseases specific to selenium-deprived populations did not produce any relevant results, there were no studies identified that fit this point and otherwise met the criteria to be included. Because of this, this criterion can effectively be ignored for the remainder of this review and is included above for completeness only.

Additionally, the exclusion point on “combination therapy treatments” necessitated further specification, as it was intended to not apply to treatments where selenium was combined with only the baseline standard of care for the thyroid conditions discussed (eg. Levothyroxine). Rather, it was intended to address combinations of treatments where each is beyond the standard of care and for which their effects are also under investigation, whether allegedly separate from selenium's effects or not. These cases would make the non-selenium addition confounding to the information sought by this review. This item was a common reason for moving records back to the include list after the AI title sorting had excluded them. If this study were to be repeated in the future, this point should be written more specifically into the text of the criterion.

And lastly, during the review process it was noted that the lab value examples originally included in our dependent variable criteria did not match those discussed in the literature as identically as expected. The lab values actually identified in our literature search were as follows:

Thyroid-specific measures: FT3, FT4, Levothyroxine (LT4), TSH, Antibody titers: TPOAb.

AITD-related cytokines: INF-γ, IL-1β.

Notably, these items do still fit our original specifications and thus still qualify in our original definitions and inclusion/exclusion criteria as well. Also of note, this was NOT a common reason for moving titles between the include and exclude lists provided by the AI title sorting, as the program had accurately identified the labs values as qualifying or not, regardless of whether we had listed them as possible examples. However, perhaps most notably, many of the included studies were not concerned with the specific values related to each disease state so much as simply the presence or absence of the disease state alone. Therefore, presence or absence of referenced disease states - HT, GD, hypothyroidism, hyperthyroidism, and Chronic lymphocytic thyroiditis (CLT) - is discussed several times in this review as essentially synonymous to our described dependant variable of “improvements” (vs. no improvements) in disease status, per the understanding that the definitions of these specific disease states are standardized and widely understood.

## 4 Data analysis

For the data analysis portion of this review, each of the final 14 papers were analyzed for their study design, population of interest, and conclusions. Then the papers were summarized per any associations the studies found between the autoimmune thyroid conditions or their evaluation parameters and low selenium levels/selenium deficiency, low selenium intake/exposure, or selenium supplementation, as well as whether these associations were significant. These results were then compiled and interpreted as discussed below to examine the statistical significance (p-values, confidence intervals) of their correlations where applicable, as well as their broader clinical relevance in the context of autoimmune thyroid disease management, and for which patients, with which measured parameters, it may be beneficial.

## 5 Results

1642 records were initially identified via PubMed, SCOPUS, Cochrane Library, and CINAHL database searches. Repeats were removed, leaving 724, then records prior to 2021 were removed, leaving 182. After title review, 48 records remained. After abstract review, 24 records remained. After a full text review, 14 records remained and were included in this review.

By reviewing the papers, we observed the association between selenium status and various thyroid disorders. Our review included several geographic locations and populations and focused on studies that related to thyroid conditions including HT, GD, and other AITDs.

The study by Cinemre et al. explored HT incidence in female patients and discovered a significant negative correlation with selenium levels (p = 0.002), indicating a potential role of selenium deficiency in HT's pathogenesis [4]. Davcheva et al. investigated AITD in Bulgarian patients and states a significant negative correlation between selenium levels and hyperthyroidism (p < 0.001) [5]. However, no correlation was noted in cases of euthyroid AITD or hypothyroidism, suggesting a complex role of selenium across different thyroid conditions [5]. Halawa et al. examined the relationship between GD and selenium status and saw a significant negative correlation (p < 0.05) [6]. However, this correlation did not extend to Graves' ophthalmopathy, implying a specific role of selenium in GD [6]. Heidari and Sheikhi investigated AITD in an iodine-sufficient area in eastern Iran and found a significant negative correlation (p < 0.001) with selenium deficiency [7].

Hu et al. investigated HT, specifically analyzing the effect of selenium supplementation on TPOAb levels. The study demonstrated a significant negative correlation (p = 0.001) after supplementation, suggesting a therapeutic role of selenium in autoimmune thyroid conditions [8]. Likewise, Kravchenko et al. investigated GD in the Kyiv region, also finding a significant negative correlation with selenium deficiency (p < 0.001) [9]. The studies by Kryczyk-Kozioł et al. (2021 & 2022) looked at HT in female patients from Kraków and AITD-related cytokines. Their findings showed significant negative correlations with selenium supplementation (p < 0.05) [10,11]. The study by Mahmoudi et al. however analyzed subclinical hypothyroidism but their results did not demonstrate a significant correlation with selenium supplementation, indicating that selenium's impact may vary depending on the thyroid disorder's severity or type [12].

The study by Vaivode et al. looked at AITD and they found a significant negative correlation with selenium levels in HT (p < 0.05) but not in GD, further underscoring the variability in selenium's role across different thyroid disorders [13]. Wang et al. focused on HT in patients with high vs low antibody levels at 3 months and 6 months. They found a significant negative correlation with selenium supplementation and TGAb at 6 months (p < 0.05) but not 3 months. TPOAb has a significant negative correlation at both at 3 and 6 months (p < 0.05) [14].

Wu et al. focused on a Chinese population, comparing the incidence of selenium deficiency and HT in areas with varying selenium intake. The study revealed a higher incidence of HT in selenium deficient areas (p < 0.0001) [15]. Zake et al. studied treatment-naïve Latvian patients and found a significant negative correlation with TPOAb levels in HT (p = 0.02). However, they did not find this correlation in GD. This highlights the specific influence of selenium on autoantibody levels in HT [16]. The study by Zheng et al. focused on dietary selenium intake and its relationship with HT in the US population. They found a negative correlation between dietary selenium and HT risk, proposing dietary selenium as a potential intervention for HT (p < 0.0001) [17].

## 6 Discussion

Similar to other studies on this topic, our review yielded variable results, which change depending on the specific thyroid condition and thyroid marker being studied. Our findings show that certain thyroid conditions, such as HT, may be effectively treated by selenium, however, the wide variety of causes and manifestations involved with different thyroid conditions may explain the inconsistent outcomes of selenium supplementation. Some of the conditions we looked at, such as subclinical hypothyroidism, were not associated with changes in selenium supplementation, which was found by Mahmoudi et al. Other conditions, such as clinical hypothyroidism, were significantly associated with low selenium supplementation. This suggests that selenium levels may be different based on the severity of HT (subclinical vs clinical). Plasma selenium level was low in hypothyroidism and AITD, but one study found that selenium was not low in GD [13]. In other studies, however, markers of selenium status suggest much lower levels of selenium in patients with GD compared with healthy controls. Though such markers did not show any similar correlation with severity of GO, nor were they significantly different between patients with active vs inactive GO [6].

An important consideration in the interpretation of selenium supplementation studies is the baseline selenium status of the population being studied. Some studies indicate that selenium supplementation serves primarily as a corrective measure for selenium deficiency, acting as a necessary replacement for an essential trace element. Other studies suggest that selenium exerts therapeutic effects irrespective of baseline levels, potentially functioning as a pharmacological agent rather than merely as a deficiency correction. This distinction is particularly relevant when comparing populations residing in selenium-rich versus selenium-poor areas. The variations in study outcomes highlight the need for more precise clinical guidance regarding patient selection for selenium supplementation, emphasizing the role of baseline selenium levels in treatment decisions.

Selenium most likely comes into play for the thyroid during metabolism of thyroid hormone and the conversion of T4 into T3. It also protects the thyroid gland from oxidative stress through its function in glutathione peroxidases and thioredoxin reductases. Furthermore, selenium may modulate immune responses, which contributes to its protective effect in autoimmune thyroid conditions. The effect of selenium supplementation may depend on the amount of time it is given for, as suggested by the findings in the paper by Wang et al. which showed different findings at – 3 vs 6 months [14]. Selenium levels also may affect lab values but not clinical disease status [16]. Also, as Wu et al. discussed, there is a worldwide variability of selenium content in soil and an associated correlation between the low selenium intake in more deficient areas and certain thyroid conditions, particularly HT, suggesting that public health efforts involving selenium supplementation would be most of interest and likely to have the most benefit in low-selenium areas and their more selenium-deficient populations [15]. Wu et al. cite particularly that large swaths of Europe, Asia, and Africa have notably lower soil content than is typical for most of North America and South America, and other larger swaths of Asia, highlighting these areas as potential strong targets for public health interventions and medical treatment modalities aimed at supplementing with selenium [15]. This may be accomplished by providing selenium rich foods. Physicians can potentially incorporate these nutritional considerations into patient specific treatment plans.

## 7 Limitations and strengths

The data base of this review was limited due to a lack of studies that evaluated selenium status as connected to dietary intake. Very few of the identified studies investigated correlations between the dietary intake and selenium status of the AITD patients, rather they were more likely to correlate status with AITD presentation, without investigating contributing factors to that status. This disconnect suggests that for future studies to better establish this connection they will likely require a separate investigation into the literature on the correlation between selenium intake and selenium status, without the narrow focus on AITD patients. However, it would be more ideal for future RCTs to specifically investigate the diet/status connection in the AITD patient group specifically. This is preferable because we cannot credibly attribute differences in selenium status between AITD patients and the study controls diets without investigations that explicitly address these aspects.

Additionally, the included studies typically relied only on lab values to measure selenium status. While this was one of this review's acceptable parameters, it does suggest that further studies are needed to correlate AITD morbidity – eg. the clinical presentations, related secondary conditions, patient subjective reporting, etc. as described in our variable definitions - with selenium intake. These factors are all typically much more directly linked to the patients' quality of life than the lab values alone and would provide a more complete picture of selenium's role in the presentation and treatment for AITDs to inform future treatment protocols. Ideally, each of these factors and their correlations with changes to dietary intake of selenium by AITD patients would be investigated individually in future studies.

The strengths of this review were that a robust number of primary studies were identified as relevant and credible and were compiled to update/inform the ongoing narrative in this subject area. Additionally, this review's results in many ways corroborated the results observed in prior reviews with updated data, contributing to the consistency and depth of understanding in an area of research with comparatively limited data. Additionally, this review's results were compiled such that they can inform future studies, by identifying gaps in the literature and clinically relevant opportunities for future investigations.

## 8 Conclusions

The papers used in this systematic review collectively suggest an inverse relationship between selenium status and severity of various thyroid disorders. This shows promise for selenium supplementation as a treatment modality for thyroid disorders, especially in HT. Further research however is required to elucidate the varied role of selenium with regards to the specific thyroid condition and patient demographics. Selenium plays a crucial role in thyroid hormone metabolism and immune function. As an essential component of selenoproteins, including glutathione peroxidases and thioredoxin reductases, selenium contributes to antioxidant defense mechanisms that protect thyroid cells from oxidative stress. Additionally, selenium-dependent deiodinase enzymes are critical in converting T4 into the more active T3, directly influencing thyroid hormone regulation. In autoimmune thyroid diseases, selenium has been implicated in modulating immune responses, with studies suggesting that supplementation may reduce levels of TPOAb and improve overall thyroid function. However, the precise mechanisms through which selenium exerts its effects remain a subject of ongoing research, necessitating further investigation to determine its full therapeutic potential in thyroid disease management. Studies combining selenium and more treatments may uncover synergy with other types of compounds. While selenium supplementation shows promise in certain thyroid conditions and patient populations, future studies focusing on different dosages, different patient demographics and health status in thyroid disease treatment intervention statuses will help describe its effectiveness and the best ways to utilize it. Due to the multifaceted nature of the findings of selenium on thyroid conditions, it may be pertinent to create individualized treatment modalities for patients based on their condition and specific needs.

During the preparation of this work the authors used Chat GPT in order to organize the study titles in preparation for an efficient and meticulous review process. After using this tool/service, the authors reviewed and edited the content as needed and take full responsibility for the content of the publication.

## CRediT authorship contribution statement

**Abhinav Nannapaneni:** Writing – review & editing, Writing – original draft, Supervision, Software, Methodology, Investigation, Formal analysis, Data curation, Conceptualization. **Victoria Wilson:** Writing – review & editing, Writing – original draft, Validation, Supervision, Resources, Methodology, Investigation, Formal analysis, Data curation, Conceptualization. **Shahzaib Chughtai:** Writing – review & editing, Writing – original draft, Software, Resources, Methodology, Investigation, Formal analysis, Data curation, Conceptualization.

## Funding statement

No funding was received for this work.

## Declaration of competing interest

The authors declare that they have no known competing financial interests or personal relationships that could have appeared to influence the work reported in this paper.

## References

[1] Deischinger Krebs, Kautzky-Willer (2022). Dietary supplements and the thyroid—an update on micronutrient supplementation. Austrian Journal of Clinical Endocrinology and Metabolism.

[2] Zuo Li, Gu, Lei (2021). The correlation between selenium levels and autoimmune thyroid disease: a systematic review and meta-analysis. Ann. Palliat. Med..

[3] Giammanco Mazzola, Giammanco Venturella Tomasello, Carini (2023). Selenium and autoimmune thyroiditis. EuroMediterranean Biomedical Journal.

[4] Cinemre Cinemre, Serinkan Degirmencioglu, Bahtiyar, Aydemir (2022). The role of selenium, selenoproteins and oxidative dna damage in etiopathogenesis of hashimoto thyroiditis. Journal of Elementology.

[5] Davcheva Kirova, Miteva Tzvetkova, Orbetzova Nonchev, Simitchiev, Kmetov (2022). Serum selenium concentration in patients with autoimmune thyroid disease. Folia Med. (Plovdiv).

[6] Halawa Abo-Shadi, Moustafa Elkateb, Marawan (2022). Study of selenium status in grave's disease patients. Egyptian Journal of Hospital Medicine.

[7] Heidari Sheikhi (2022). Serum selenium status in Graves' disease and Hashimoto's thyroiditis in an iodine-sufficient area: a case–control study [Article]. J. Res. Med. Sci..

[8] Hu Feng, Chen Shi, Jiang Zheng, Liu Zhang, Ge Liu (2021). Effect of selenium on thyroid autoimmunity and regulatory T cells in patients with Hashimoto's thyroiditis: a prospective randomized-controlled trial [Journal article]. Clinical and translational science.

[9] Kravchenko Grossman, Rakov Kovzun, Pankiv, Simurov (2021). Selenium supply and thyroid condition in Graves' disease in the region of iodine deficiency. Problemi Endokrinnoi Patologii.

[10] Kryczyk-Kozioł Zagrodzki, Prochownik Błażewska-Gruszczyk, Słowiaczek Sun, Schomburg Ochab, Bartyzel (2021). Positive effects of selenium supplementation in women with newly diagnosed Hashimoto's thyroiditis in an area with low selenium status. Int. J. Clin. Pract..

[11] Kryczyk-Kozioł Prochownik, Błażewska-Gruszczyk Słowiaczek, Sun Schomburg, Ochab Bartyzel, Zagrodzki (2022). Assessment of the effect of selenium supplementation on production of selected cytokines in women with Hashimoto's thyroiditis. Nutrients.

[12] Mahmoudi Mobasseri, Ostadrahimi Pourmoradian, Soleimanzadeh, Kafili (2021). Effect of selenium-enriched yeast supplementation on serum thyroid-stimulating hormone and anti-thyroid peroxidase antibody levels in subclinical hypothyroidism: randomized controlled trial. Adv. Biomed. Res..

[13] Vaivode Zake, Strele Upmale-Engela, Gogins Gersone, Skesters Dambrova, Konrade (2023). Stress-related immune response and selenium status in autoimmune thyroid disease patients. Int. J. Mol. Sci..

[14] Wang Sun, Li, Wang (2021). The effects of selenium supplementation on antibody titres in patients with Hashimoto's thyroiditis. Endokrynol. Pol..

[15] Wu Wang, Chen Wei, Lv Wang, Wu Zhao, Peng Rijntjes, Wang Schomburg, Shi (2022). Increased incidence of Hashimoto thyroiditis in selenium deficiency: a prospective 6-year cohort study. J. Clin. Endocrinol. Metab..

[16] Zake Kalere, Engela Upmale, Svirskis Gersone, Skesters Groma, Konrade (2021). Plasma levels of Th17-associated cytokines and selenium status in autoimmune thyroid diseases. Immun Inflamm Dis.

[17] Zheng Cai, Guo Song, Hu Li, Zhu (2023). The association between dietary selenium intake and Hashimoto's thyroiditis among US adults: national Health and Nutrition Examination Survey (NHANES), 2007–2012. JEI (J. Endocrinol. Investig.).

